# Fear extinction learning modulates large-scale brain connectivity

**DOI:** 10.1016/j.neuroimage.2021.118261

**Published:** 2021-06-11

**Authors:** Zhenfu Wen, Zhe Sage Chen, Mohammed R. Milad

**Affiliations:** a Department of Psychiatry, New York University School of Medicine, New York, NY, United States; b Department of Neuroscience and Physiology, New York University School of Medicine, New York, NY, United States; c The Neuroscience Institute, New York University School of Medicine, New York, NY, United States; d Nathan Kline Institute for Psychiatric Research, Orangeburg, NY, United States

**Keywords:** Learning and memory, Fear conditioning, Brain activation, Neuroimaging and plasticity

## Abstract

Exploring the neural circuits of the extinction of conditioned fear is critical to advance our understanding of fear-and anxiety-related disorders. The field has focused on examining the role of various regions of the medial prefrontal cortex, insular cortex, hippocampus, and amygdala in conditioned fear and its extinction. The contribution of this ‘fear network’ to the conscious awareness of fear has recently been questioned. And as such, there is a need to examine higher/multiple cortical systems that might contribute to the conscious feeling of fear and anxiety. Herein, we studied functional connectivity patterns across the entire brain to examine the contribution of multiple networks to the acquisition of fear extinction learning and its retrieval. We conducted trial-by-trial analyses on data from 137 healthy participants who underwent a two-day fear conditioning and extinction paradigm in a functional magnetic resonance imaging (fMRI) scanner. We found that functional connectivity across a broad range of brain regions, many of which are part of the default mode, frontoparietal, and ventral attention networks, increased from early to late extinction learning only to a conditioned cue. The increased connectivity during extinction learning predicted the magnitude of extinction memory tested 24 h later. Together, these findings provide evidence supporting recent studies implicating distributed brain regions in learning, consolidation and expression of fear extinction memory in the human brain.

## 1. Introduction

Fear extinction refers to the decrement in conditioned fear responses after unreinforced presentation of a conditioned stimulus. Abnormal fear extinction is thought to characterize fear and anxiety-related disorders, such as posttraumatic stress disorder (PTSD) and anxiety disorders (Craske et al., 2017; Fenster et al., 2018; Ressler, 2020). Therefore, understanding the neural mechanisms of fear extinction will advance our understanding of the underlying psychopathology of these disorders.

During the past two decades, neural circuits of fear extinction have been extensively studied using animal models and then translated to humans (Herry et al., 2010; Milad and Quirk, 2012). Partially inspired by animal studies, previous human neuroimaging explorations mainly focus on a so-called ‘fear network’, including the ventromedial prefrontal cortex (vmPFC), dorsal anterior cingulate cortex (dACC), insular cortex, hippocampus, amygdala (Milad and Quirk, 2012; Picó-Pérez et al., 2019; Ressler, 2020). These regions are largely involved in mediating fear, arousal, threat-detection, and regulating responses to fearful and conditioned stimuli (Etkin and Wager, 2007). Studies focusing on these regions have advanced our understanding of fear and its regulation. However, this ‘fear network’ view does not fully take into consideration the potential role of higher cortical regions in learning and memory. Recent meta-analyses focusing on brain activations during fear conditioning and extinction revealed the engagement of multiple cortical regions during the fear conditioning and extinction tasks (Fullana et al., 2016, 2018). A recent study conducted by Berg et al. showed that activations across well-defined networks including the salience, and the default mode networks are engaged in a fear generalization task, going beyond the limited focus on the traditional ‘fear network’ (Berg et al., 2020). The additional cortical regions with in the salience, ventral attention, and frontoparietal networks might play a critical role in the multiple tasks required during extinction learning, like conscious awareness of the cues, attention allocation, perceptual processes, and working memory load during the task–processes that are needed for enabling the subjective feeling of fear (LeDoux and Pine, 2016). The data from these recent studies implicating a broader range of cortical regions in fear extinction learning and recall is consistent with a recent call by LeDoux and Pine for an extended framework to study fear and anxiety- termed a “two systems” framework (LeDoux and Pine, 2016). In such, the first system responds to threat, and then generates behavioral and physiological reactions in a non-conscious manner. The second system allows conscious feelings of the threat and the volitional regulation of reactions (LeDoux and Pine, 2016).

It is well documented that extinction learning leads to the formation of a new memory (Milad and Quirk, 2012; Sevenster et al., 2018). But this memory is a complex one—requiring not just the formation of the new association (that is, a conditioned cue no longer predicts the unconditioned stimulus), but also entails conscious awareness, attention, and recollection of several elements of the learning experience, including the color and shape of the cue, the context, how the experiment was conducted, timing between cues, etc. Where is all that information stored? Prior studies have mainly examined the interactions between nodes of the 'fear network' with a primary focus on the amygdala, hippocampus, and the vmPFC in the learning, consolidation, and expression of the extinction memory, suggesting that extinction memory resides within these regions. However, memory storage, especially for complex learning and memory paradigms is unlikely to reside within a given brain region or within a few. The classic work of Karl Lashley (Lashley, 1950) concluded that memory engrams (the term 'engram' was first introduced by Richard Semon in 1904 to refer to the physical trace of a memory (Josselyn and Tonegawa, 2020)) are not likely localized to a discrete cortical region (Lashley, 1950). More recent literature suggest that structural representations of memory engrams are distributed across the brain, including both subcortical and cortical areas (Josselyn et al., 2015; Tonegawa et al., 2015; Josselyn and Tonegawa, 2020). This is well-aligned with the current view that brain function depends on highly complex interactions between distributed brain regions (Sporns, 2014; Braun et al., 2018). Some preliminary studies, though not in the context of fear learning, have shown that learning will extensively reorganize brain activation and connectivity (Bertolero et al., 2020). Together, these data suggest that broadly distributed brain regions are likely engaged during the extinction of conditioned fear responses.

Exploring how extinction learning modulates local regional activity and functional connectivity between remotely distributed regions would thus enhance our understanding of its underlying mechanisms. In this study, we analyzed fMRI data from 137 healthy participants who underwent a two-day fear conditioning and extinction paradigm. Going beyond the traditionally-defined “fear network”, we explored how fear extinction learning and memory expression modulate whole-brain connectivity and activation. Considering that extinction learning is intrinsically dynamic, we conducted our analysis in a trial-by-trial manner, such that to enable us to track the gradual modulations in connectivity and activation associated with learning and plasticity over the course of fear extinction. We further explored how these modulations correlate with the follow-up extinction memory retrieval. We predicted the engagement (as evident by increased connectivity and activation) of a large number of brain regions, especially within the frontal and parietal cortices during the later phase of extinction learning. We also predicted that the magnitude of increased networks connectivity during extinction learning would correlate with the magnitude of extinction memory recall when tested after a delay. We observed a significant increase in functional connectivity during late extinction learning in approximately 30% of the brain; the strength of which predicted the magnitude of extinction memory tested 24 h later. Contrary to our prediction, however, we observed a disassociation between functional activation and functional connectivity during extinction learning.

## 2. Materials and methods

### 2.1. Participants

A total of 137 healthy individuals (94 females) with normal or corrected-to-normal vision participated in the study. The data used in this manuscript had been used in prior studies focusing on other questions related to fear extinction and had been previously published (Milad et al., 2009; Marin et al., 2016, 2020). All procedures were approved by the Institute Review Board (IRB) of the Massachusetts General Hospital, Harvard Medical School. Written informed consent was obtained from all participants.

### 2.2. Experimental design

All participants underwent a two-day fear conditioning and extinction paradigm in an fMRI scanner. The detailed paradigm has been described previously and is briefly described below (Milad, Quirk, et al., 2007, 2007; Milad et al., 2008, 2009; Marin et al., 2016). The paradigm consisted of several phases (Figure S1), and the trial structure was identical across different phases. Each trial started with a blank screen lasting 12–18 s (mean: 15 s), followed by a picture of a room (either a library or an office) with an unlit lamp for 3 s (context) after which the lamp turned on to blue, red or yellow and lasted for 6 s (conditioned stimulus, CS). On the first day, participants were instructed to select their level of electric stimulation to be used during the experiment, so that the stimulation level was highly annoying yet non-painful. Selected shock levels ranged from 0.6 to 4.0 mA across participants. Electrical stimulation was delivered through electrodes that were attached to the index and middle finger of the participanťs dominant (right) hand. The paradigm then started with a habituation stage in which all paradigm images were presented to the participant, without any electrical stimulation. Following the habituation, fear conditioning occurred in one context (e.g., the 'office' picture) where two of the colored lamps (e.g., blue and red, CS+) were partially reinforced (62.5% reinforcement rate) with a mild electric shock (500 ms, occurring at the offset of CS presentation) and the other colored lamp (e.g., yellow) was never paired with the shock (CS-). Fear conditioning consisted of a total of 32 trials (8 presentations for each of the two CS+s and 16 presentations of CS-). Following this, extinction learning occurred in a different context (e.g., the library), where one of CS+ was presented 16 times without any shocks (CS+ extinguished; CS+E) intermixed with 16 presentations of CS-. On the next day, extinction memory recall was tested in the context of extinction learning (e.g., the 'library' picture), where the three colored lamps were presented (8 presentations of the extinguished CS+E, 8 presentations of the unextinguished CS+ (CS+U) along with 16 presentations of CS-). The order of stimulus presentation was pseudo-random for all stages of the paradigm

### 2.3. Psychophysiology

Skin conductance response (SCR) was measured throughout the scanning session using MRI-compatible electrodes. SCR was scored as previously described (Milad, Wright, et al., 2007; Marin et al., 2017, 2020). For each phase of the paradigm (conditioning, extinction, and recall), conditioned responses for each trial were calculated by subtracting the average skin conductance level during the last 2 s of the context presentation from the maximum skin conductance level during the CS presentation. All SCR analyses were performed on square-root transformed data.

### 2.4. MRI acquisition and preprocessing

Human neuroimaging data were acquired in a Trio 3.0 Tesla whole-body MRI scanner (Siemens Medical Systems, Iselin, NJ) using a 32-channel head coil. Functional data were acquired using a T2*-weighted echo-planar imaging (EPI) pulse sequence (TR: 2.56 s, TE: 30 ms, slice number: 48, voxel size: 3 × 3 × 3 mm). Anatomical images were acquired using a T1-weighted MPRAGE pulse sequence (TR:2300 ms, TE: 3.03 ms, 192 sagittal slices, voxel size: 1 × 1 × 1 mm). Preprocessing was performed using fMRIPrep 20.0.2 (Esteban et al., 2019). Functional images were corrected for slice timing, realigned, co-registered with the structural image, normalized into MNI space, and smoothed with an 8-mm full width half-maximum Gaussian kernel.

### 2.5. MRI data analysis

We explored the dynamics of whole-brain connectivity across multiple experimental phases. With this approach, we first obtain the activation values of a given brain region in response to the presentation of a CS across an experimental phase. The correlation between activation series of this region and every other region within the brain is then calculated. The resulting brain map becomes a depiction of increased ('hoť colors) or decreased ('cool' colors) task-induced functional connectivity at the whole brain level. To accomplish this, we estimated the brain connectivity by combining a beta series correlations method (Rissman et al., 2004) and a jackknife correlation method (Miller, 1974; Richter et al., 2015), such that we could measure the relative difference in connectivity at each single trial during learning and recall (i.e., CS+ or CS-) compared to all other trials (Thompson et al., 2018).

We estimated voxel-wise activation by using the Generalized Linear Model (GLM) approach implemented within the SPM12 toolbox (https://www.fil.ion.ucl.ac.uk/spm/software/spm12/). For the extinction and recall phase of each subject, we used the least-squares-all (LSA) model approach to estimate brain activation for each single trial (Mumford et al., 2012). Specifically, the model included 32 regressors for each of the CS presentation and one regressor for the context presentation. All regressors were modeled as boxcar functions and convolved with the canonical hemodynamic response function. We also added motion parameters from the realignment process, used high-pass temporal filtering (128 s) and a first-order autoregressive (AR) model to account signal drift and biorhythms.

After the single-trial GLM estimation, we obtained 16 CS+ and 16 CS- beta images for the extinction phase, 8 CS+*E*, 8 CS+*U* and 16 CS-beta images for the recall phase. For each beta image, we extracted regional beta value by averaging beta values of voxels from each brain region. In this study, we have used a whole-brain parcellation consisting of 400 cortical regions (Schaefer et al., 2018) and 32 subcortical regions (Tian et al., 2020). Thus, we obtained a 432-dimensional beta vector to represent regional activation for each trial. We further z-scored each element of the vector across all trials of each experimental phase, so that each element represented relative activation across the experiment.

We adopted the beta series correlations (BSC) method (Rissman et al., 2004) to estimate pair-wise connectivity among brain regions. The BSC method calculated the correlation between condition-specific beta series of two regions to represent their connectivity. To further obtain single-trial connectivity estimation, we employed a jackknife approach (Miller, 1974). The jackknife correlation has been successfully applied to capture single-trial coherence on electrocorticographic data (Richter et al., 2015), and time-varying connectivity on resting-state fMRI data (Thompson et al., 2018). In this study, the jackknife correlation procedure was conducted as follows. First, we left out the beta vector of one specific trial; Second, we calculated a pair-wise regional Pearson's correlation based on the remaining 31 beta vectors to obtain a 432 × 432 connectivity matrix; Third, we repeated the above two steps for each of the 32 trials to obtain 32 connectivity matrices, and z-scored each element of the matrix across trials; Fourth, we averaged each connectivity matrix across the row (with the diagonal elements excluded) to obtain a 432-dimentional vector to represent the regional connectivity, with each element of this vector summarizing connectivity strength between a specific region and all other regions (Buckner et al., 2009; Cole et al., 2013). Both negative and positive correlations were included in calculating the regional connectivity. This choice was made based on previous studies on global functional connectivity (Cole et al., 2010; Tagliazucchi et al., 2016; Preller et al., 2018). And also based on the fact that the jackknife correlation estimates the relative functional connectivity across trials, i.e., a negative correlation in a trial means the correlation value in this trial is relatively smaller than in other trials, but not that the two regions are fluctuating in opposite directions. Therefore, in a trial, a region with high regional connectivity means that the activity of this region is strongly correlated to the rest of the brain compared to other trials. Finally, we reordered all trials according to CS type (e.g., CS+ and CS-) and presentation time (e.g., from 1st to 16th CS+ trial) during the experiment.

For the extinction learning phase, we compared the connectivity (or activation) difference between paired CS+ and CS- for each brain region (e.g., the 1st CS+ minus 1st CS-). As in our previous studies, we divided the 16 paired CS+ vs. CS- trials into 4 time-blocks (from 1 to 4, representing early to late extinction learning). We performed regional significant tests (CS+ vs. CS-) on each time-block as reported in previous studies (Milad, Wright, et al., 2007; Rabinak et al., 2014; Marin et al., 2017). We also conducted network-based statistic analyses (Zalesky et al., 2010) to identify connections that showed significant differences for extinction learning CS+ vs. CS- (Figure S2, see Supplemental Material for details). For the recall phase, we focused on the first 4 trials of CS+E and its paired CS-. We divided the 4 paired CS+E vs. CS- trials into 2 time-blocks (early and late extinction recall). We also analyzed the data for the CS+U; the results can be found in Supplementary Material (Figure S3).

### 2.6. Correlation analyses

We examined the correlation between brain connectivity (or activation) in the extinction learning phase and the extinction retention index (ERI), a validated psychophysiological measure used as an index of extinction memory (Milad et al., 2009; Hartley et al., 2011; Raio et al., 2014; Raij et al., 2018): the higher the ERI, the better the extinction learning (but see (Lonsdorf et al., 2019)). The magnitude of ERI was calculated as follows: each subjecťs average SCR for the first 2 CS+E trials during the extinction recall phase was divided by their largest SCR to a CS+E trial during the conditioning phase and then multiplied by 100, which yielded a percentage of maximal conditioned responding. This percentage was subtracted from 100% to yield the ERI.

We conducted correlation analyses on brain regions that showed significant connectivity differences (CS+ vs. CS-) in the extinction learning phase. We first examined whether the overall change of mean connectivity/ activation differences across regions from early to late extinction learning phase (defined as [late CS+ – late CS-] – [early CS+ – early CS]) correlated with the ERI. To further explore the dynamic relationship between regional signals and ERI, we also examined the correlation between the ERI and regional connectivity or activation differences (CS+ – CS-) in each time-block.

### 2.7. Significant test

Weused non-parametric paired statistics (Wilcoxon signed rank test) to compare regional connectivity or activation differences (CS+ vs. CS-) across subjects. We performed false discovery rate based (FDR-based) correction for multiple comparisons. We used Pearson’s correlation to measure the correlation between the ERI and neural data, and used bootstrap to estimate the confidence interval.

## 3. Results

### 3.1. Modulation of brain connectivity during fear extinction learning and memory recall

**Fig. 1.**
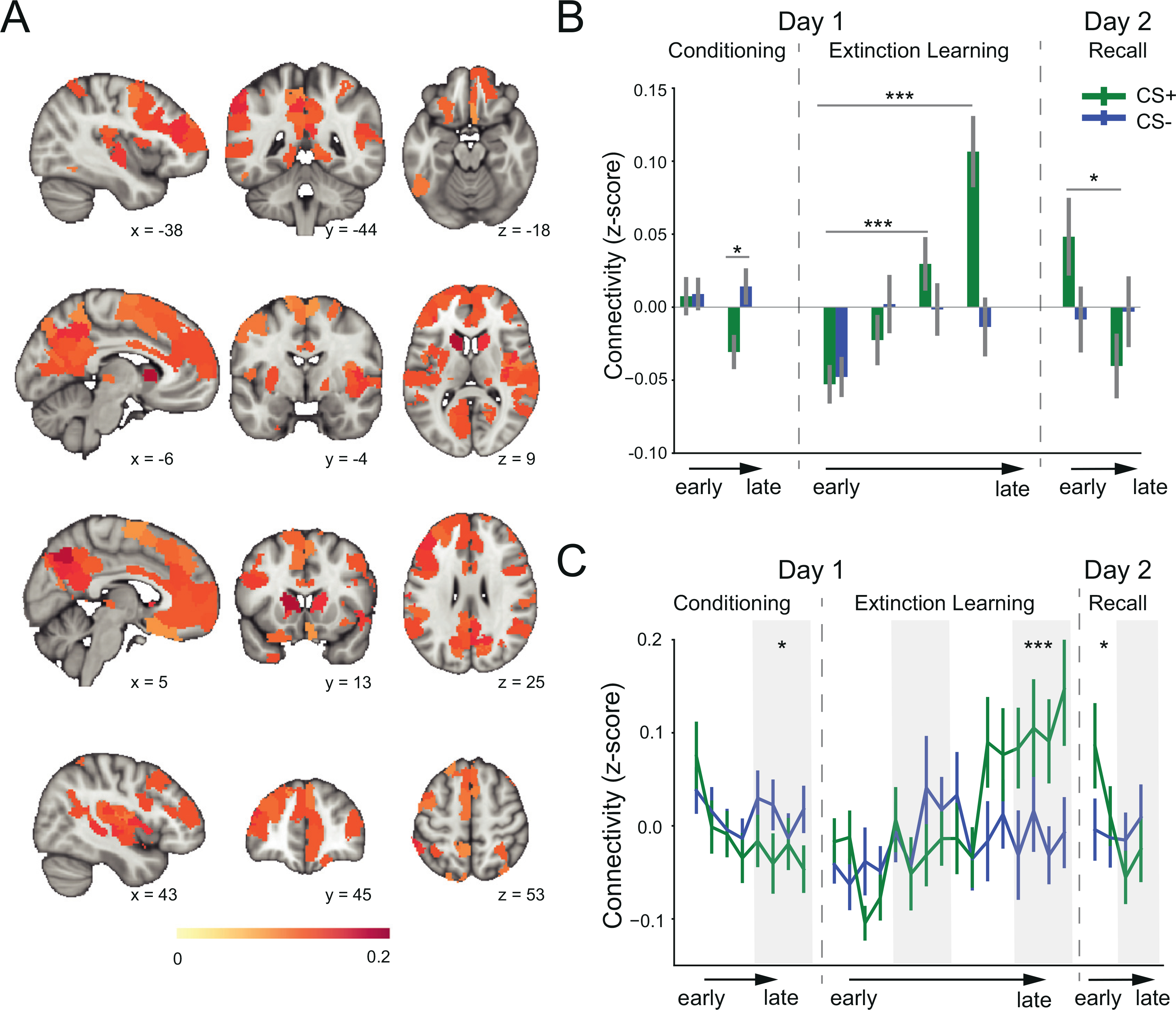
Brain connectivity changes during fear conditioning, extinction learning, and memory recall. A. Brain regions that exhibited significant difference in regional connectivity (CS+ minus CS-) during late extinction learning. B. Mean connectivity in time-blocks for all brain regions showing significant extinction learning-induced connectivity. In recall phase, the CS+ represents CS+E. C. Single-trial level mean connectivity across the significant regions. *: *p <* 0.05, * *: *p <* 0.01,***:*p <* 0.001.

**Fig. 2.**
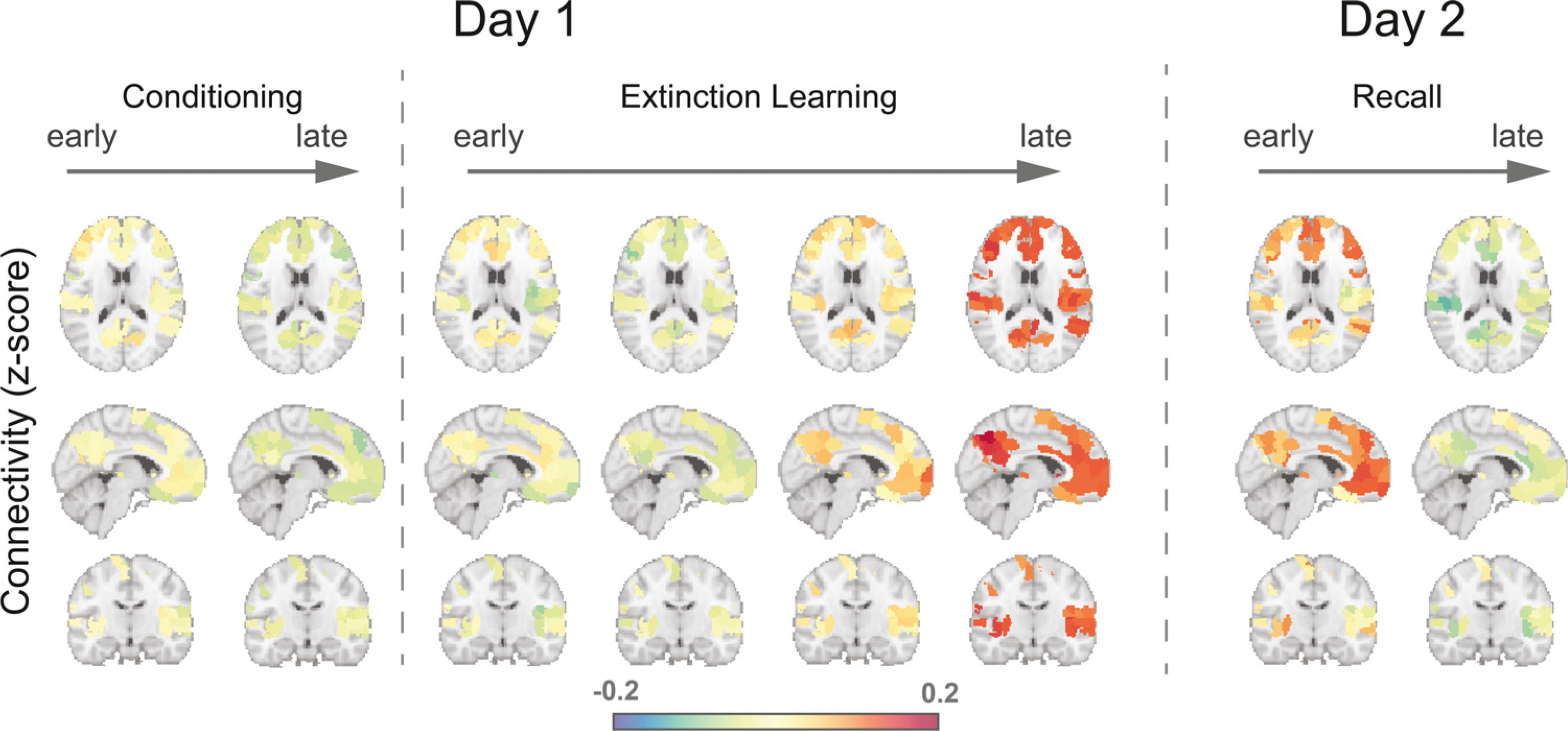
Brain regions displaying altered connectivity patterns across experimental phases. In day 1, brain maps represent regional connectivity differences between CS+ and CS-. In day 2, brain maps represent regional connectivity differences between CS+E and CS-.

We first explored the dynamics of whole-brain connectivity across the extinction learning and early recall phases. We conducted a time-block x CS-type 2-way repeated measures ANOVA with mean functional connectivity across whole brain as the dependent variable. This analysis revealed a significant main effect of time-lock (F(3, 408) = 8.35, *p* < 0.001), and a significant interaction between CS-type and time-block (F(3, 408) = 3.41, *p* = 0.018). This suggested that extinction learning-induced changes in brain connectivity are different for the CS+ and CS-. We then conducted significance tests on the mean regional connectivity difference for each time-block. Our analyses show that differences in connectivity between the CS+ and CS- began to emerge after approximately 8 trials of extinction learning. Statistical significance between the CS+ and CS- was noted during the last 4 extinction learning trials in 133 brain regions (Fig. 1A, *p* < 0.05, Wilcoxon signed rank test, FDR-based correction), suggesting that these regions exhibited larger connections with other regions during CS+ processing than CS- processing. We assigned anatomical labels to these regions according to their MNI coordinates and the automated anatomical labelling (AAL) atlas (Table S1). The averaged z-scores in time-blocks for all brain regions shows significant learning-induced connectivity (Figs. 1B, 2). We observed a significant difference in CS+ associated connectivity within the third time-block compared to the first time-block (t_136_ = 3.51, *p* < 0.001), and the magnitude of this difference further increased in the fourth time-block (t_136_ = 5.19, *p* < 0.001). In contrast, there was no significant difference in mean connectivity between the first and any other time-block for CS- trials (all *p* > 0.10). Fig. 1C shows the trial-by-trial changes in connectivity to display enhanced temporal resolution. These results show that extinction learning-induced changes in brain connectivity are specific to the CS+ and are induced by extinction learning roughly mid-way through extinction learning. On day 2, after extinction learning consolidation, the brain connectivity to the CS+E started high and gradually decreased from the first time-block to the second time-block of extinction memory recall (t_135_ = 2.28, *p* = 0.024), whereas the brain connectivity to the CS- trials remained at a similar level for the two time-blocks (t_135_ = 0.15, *p* = 0.88) (Figs. 1B, C). These results suggest that the brain connectivity increases observed during extinction learning were maintained overnight until memory retrieval was being tested.

We also conducted analyses to examine the connectivity changes during fear conditioning. We tracked the mean connectivity across the 133 brain regions identified in late extinction changed from early to late fear conditioning (Figs. 1B, 1C). We did not observe significant connectivity changes from early to late fear conditioning to the CS+ (t_120_ = 1.87, *p* = 0.064) or CS- (t_120_ = -0.27, *p* = 0.78) processing. But CS+ associated connectivity was lower than that of the CS- associated connectivity during late fear conditioning (t_120_ = -2.24, *p* = 0.027). This decreased connectivity in late conditioning is consistent with animal and human studies that reported decrease in neuronal activity during fear conditioning in regions implicated in fear extinction (Garcia et al., 1999; Phelps et al., 2004). We also compared the regional connectivity (CS+ vs. CS-) in early/late fear conditioning. We did not observe significant differences in early conditioning, but we found 4 regions (one in superior frontal gyrus, the others in visual cortex) that exhibited lower connections with other regions during CS+ processing relative to CS-processing in late fear conditioning (with a criterion of FDR-corrected *p* < 0.05). These results suggested that the connectivity changes during extinction learning are different from those during fear conditioning.

Do the brain regions showing significant increase in connectivity belong to well-defined/established networks related to consciousness, attention, or memory processing? To answer these questions, we assigned each region into one of 8 functional subnetworks according to previous studies (Thomas Yeo et al., 2011; Schaefer et al., 2018): the visual network (VN), somatomotor network (SMN), dorsal attention network (DAN), ventral attention network (VAN), limbic network (LMN), frontoparietal network (FPN), and the default mode network (DMN) (Thomas Yeo et al., 2011), as well as the subcortical network (SCN) (Tian et al., 2020). We then computed the percentage of regions within each subnetwork that showed significant increase in connectivity (Figure S4). The DMN, the FPN, and the VAN contained the highest percentage of brain regions that were significantly modulated by learning (DMN: 44%, FPN: 40%, and VAN: 38%). For the 5 remaining networks, 18%-27% of brain regions exhibited significant increase in connectivity. Since the regional connectivity measure is not quite specific regarding the distribution of the functional connectivity, we also conducted a network-based statistic (NBS) analysis (Zalesky et al., 2010), which allowed us to localize connections modulated by extinction learning. In late extinction learning (Figure S2), changes of connectivity were mostly between the DMN and VAN, SMN, and SCN. The connectivity between the FCN and other subnetworks also changed significantly. These results are consistent with the main analyses based on regional connectivity measure, indicating that extinction learning-induced modulations in brain connectivity are predominant in networks involved in emotion regulation, memory storage and conscious attention processing, but were not limited to a specific functional subnetwork.

### 3.2. Dissociation of connectivity and activation

**Fig. 3.**
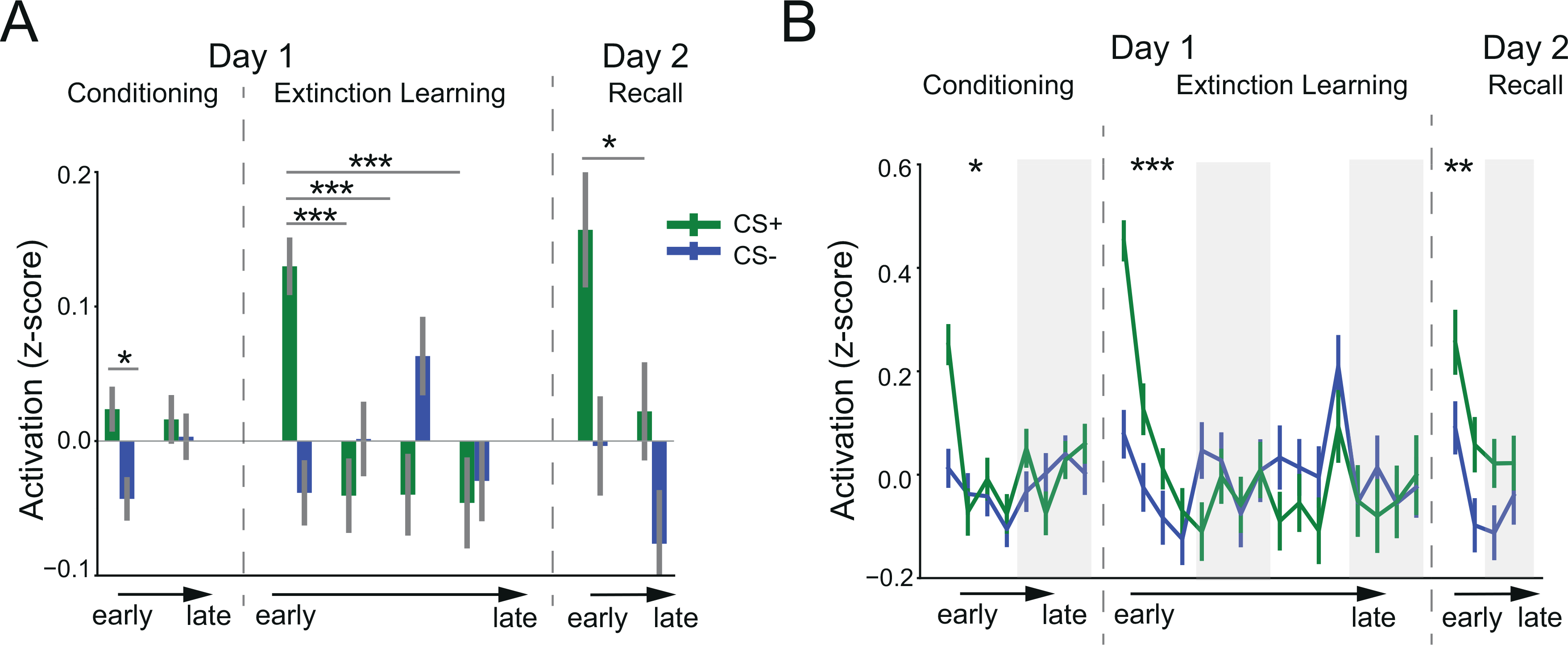
Brain activation changes during fear conditioning, extinction learning, and memory recall. A. Time-block level mean activation across the significant regions identified in late extinction learning. In recall phase, the CS+ represents CS+E. B. Single-trial level mean activation across the significant regions. *: *p <* 0.05,**: *p <* 0.01, * * *: *p <* 0.001.

**Fig. 4.**
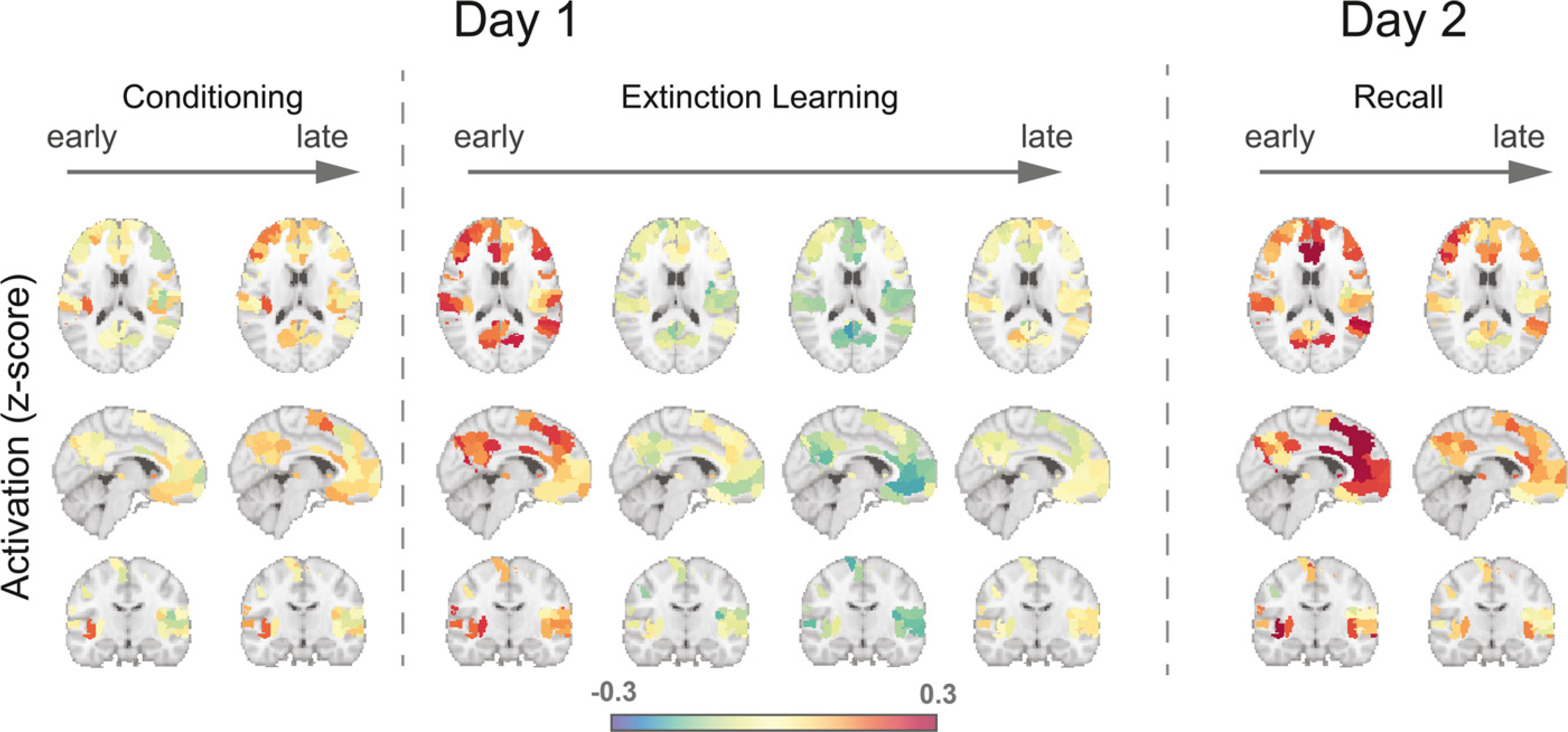
Brain activation patterns across experimental phases. In day 1, brain maps represent regional activation differences between CS+ and CS-. In day 2, brain maps represent regional activation differences between CS+E and CS-. Only regions exhibited significant connectivity differences during late extinction learning were shown.

We have shown thus far that connectivity in 133 brain regions was modified during extinction learning and memory recall. What about the functional activation of these same regions? Are the connectivity changes also associated with functional activations during the same time frame? To answer these questions, we began by examining each significant region's activation (CS+ minus CS-) across the experimental phases. During extinction learning, in contrast to their connectivity, activations of these brain regions decreased from early to late extinction trials (Figs. 3, 4). Mean activations started high in the early stage of CS+ presentation, and then dropped quickly after a few trials, with the mean activation in the first time-block significantly higher than that in the last time-block (t_136_ = 3.85, *p* < 0.001, Fig. 3A). In contrast, CS- trials exhibited significantly lower mean activation than CS+ trials in the early extinction stage (t_136_ = -5.65, *p* < 0.001). This level of mean activation remained across the extinction phase, with no significant difference between early and late stage for CS- trials (t_136_ = 0.22, *p* = 0.82). During the extinction recall phase, activation differences started high and gradually decreased from the early phase to late phase (Figs. 3A, B). There was a significant difference in mean activation between CS+ and CStrials in the first time-block of memory recall (t_135_ = 2.79, *p* = 0.006), but this difference diminished in the second time-block (t_135_ = 1.71, *p* = 0.089). Overall, these results suggested that in contrast to functional connectivity, the modulation of brain activation mainly occurred in the early stage of extinction learning and memory recall.

### 3.3. Association of extinction learning-induced connectivity and activations with memory recall

**Fig. 5.**
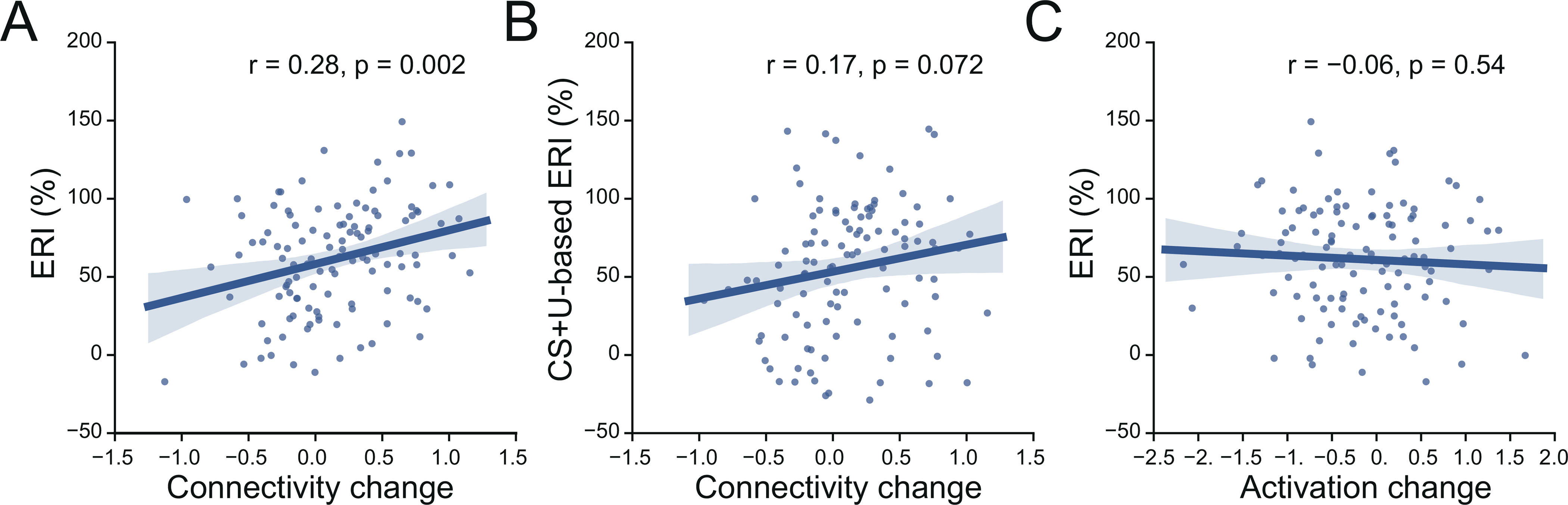
Correlations between mean connectivity and activation changes and the extinction retention index. A. Correlation between the extinction retention index (ERI) and the change in mean brain connectivity across significant brain regions. B. Correlation between the CS+U-based ERI and the change in brain activation across significant brain regions. C. Correlation between the ERI and the change in brain activation across significant brain regions.

The increase in brain connectivity from early to late extinction learning suggests that such changes may be associated with the consolidation of the extinction memory. Are these connectivity changes related to, or predictive of, the magnitude of extinction memory recall? To test such possibility, we explored whether the extinction learning-induced increase in connectivity is associated with extinction memory expression (measured with ERI) during memory recall. Correlation analyses revealed that the overall increase in mean connectivity across regions from early to late extinction learning phase was positively correlated with the ERI (Pearson's correlation *r* = 0.28, *p* = 0.002, Fig. 5A), suggesting that a larger increase in overall connectivity during extinction learning resulted in better recall of the extinction memory. We also defined the ERI by using the SCR of unextinguished CS+ in the recall phase (CS+U) and found that this CS+U-based ERI was marginally correlated with the increased connectivity (*r* = 0.17, *p* = 0.072, Fig. 5B), suggesting that the connectivity changes observed during extinction learning might also generalize, to some degree, to the cue that was not extinguished. We further examined correlations between the ERI and mean connectivity of each subnetwork. The significant correlations with ERI were observed mainly within and between subnetworks of VAN, SMN and VN (Figure S5). In contrast to functional connectivity, correlations between the CS+E-based ERI and the change in mean activation during the extinction learning phase were not significant (*r* = −0.06, *p* = 0.54, Fig. 5C).

**Fig. 6.**
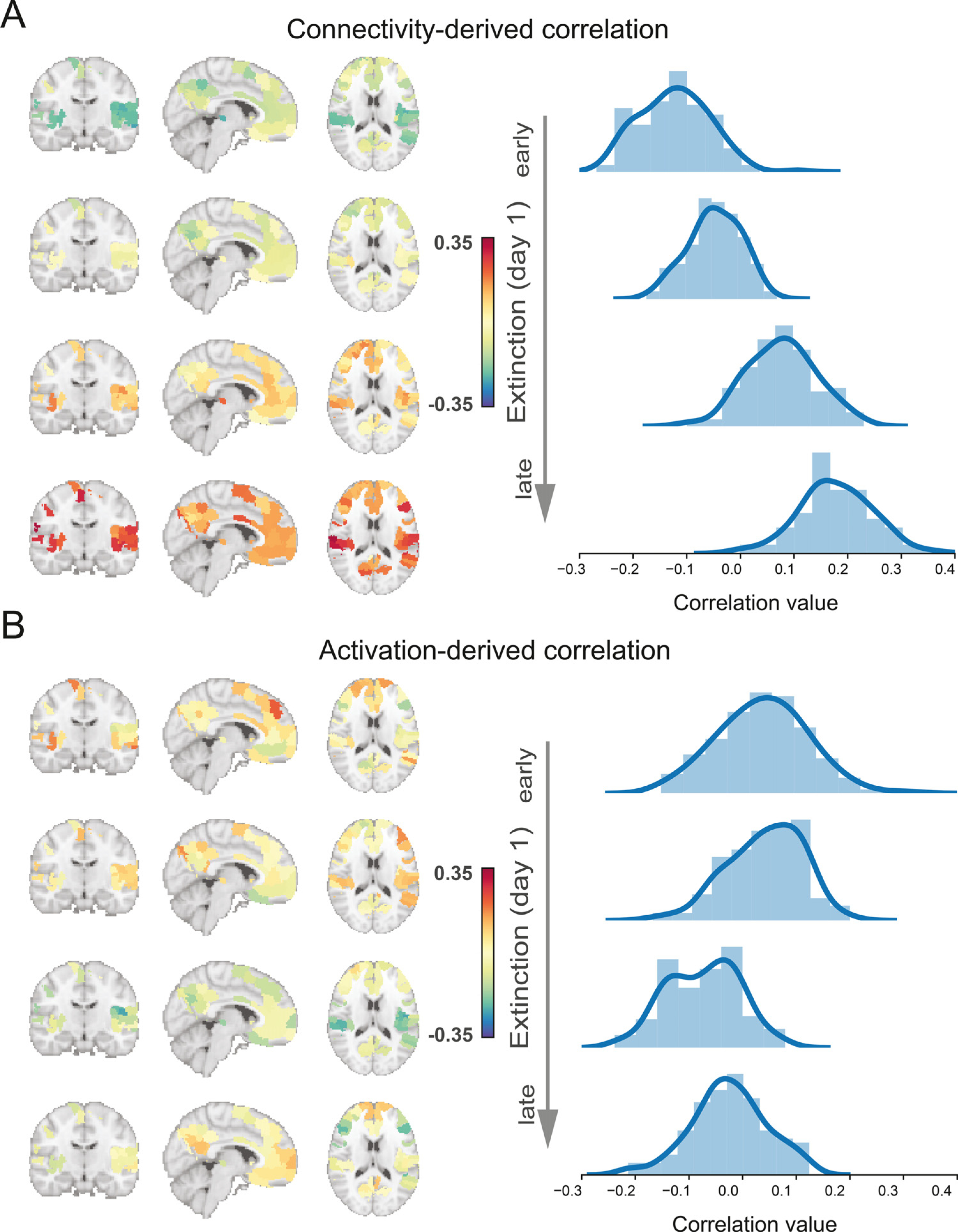
Distributions of correlations between observed connectivity and activation patterns and the extinction retention index. A. Distribution of the Pearson’s correlation value between the regional connectivity difference and extinction retention index (ERI) during four (early-to-late) extinction stages. B. Distribution of the Pearson’s correlation between the regional activation difference and ERI during four (early-to-late) extinction stages.

To further explore the temporal dynamic relationship between the ERI and regional neural signals over the extinction learning phase, we examined the correlation between the ERI and each region's connectivity/ activation differences (CS+ minus CS-) in each time-block. In terms of connectivity, the correlation histogram exhibited a shift from mostly negative correlations in early extinction learning to mostly positive correlations towards the end of extinction learning (Fig. 6A). In contrast to connectivity, the correlation between regional activation and ERI shifted from a weak positive correlation to a weak negative correlation across the extinction phase (Fig. 6B). These results suggested that neural signals in extinction learning were predictive of the magnitude of extinction memory recall and provide further support that modulations of brain connectivity and mean activation were dissociated across extinction learning.

## 4. Discussion

We explored the temporal dynamics of whole-brain connectivity and activations induced by conditioned fear extinction learning and memory recall in 137 healthy participants. We reported significant global network changes induced by extinction learning that were specific to the conditioned cue. These changes in connectivity extended far beyond the traditionally studied structures in fear conditioning and its extinction; covering diverse functional communities such as the default mode network and the frontoparietal network. The learning-induced connectivity changes were dissociated from activations. We showed that the learning-induced connectivity increases were associated with the magnitude of the extinction memory expression when tested 24 h after learning. Lastly, additional analyses within specific well-defined networks also enabled a comparable conclusion—a diffused functional connectivity across multiple networks are specific to the conditioned cue, and that learning-induced functional connectivity within and between specific networks (mainly the ventral attention, somatomotor, and visual networks) predicted the magnitude of the success of extinction memory recall tested later.

The majority of published studies to date have focused on regions commonly referred to as 'fear-related' brain circuits such as the amygdala, hippocampus, and vmPFC (Milad and Quirk, 2012; Picó-Pérez et al., 2019; Ressler, 2020). However, the results we report here show that the 'engram' of fear extinction memory involves a large network of brain circuits that extends far beyond the traditionally studied 'fear circuiť. The broader engagement of these brain regions is likely to reflect many other aspects of the extinction learning experience, including the perceptual, attention, and conscious memory associated with the conditioned stimulus. This is consistent with the view recently proposed posed by LeDoux-Pine—the “two systems” view of fear (LeDoux and Pine, 2016). Our results implicate 133 brain regions (out of a total of 432) that exhibited robust learning-induced changes in connectivity. Some were within the default mode network (DMN), such as the posterior cingulate cortex and several prefrontal cortical areas (Fig. 1A). The DMN has been implicated in conscious awareness (Raichle, 2015) and affective learning (Marstaller et al., 2017). Independent studies also suggested that the dysfunction of the DMN may contribute to the impairment of fear extinction in individuals with posttraumatic stress disorder (PTSD) (Miller et al., 2017). In addition to the DMN, connectivity changes were noted in a large scale within the frontoparietal network (FPN), which is known for playing an important role in attentional control (Scolari et al., 2015). The top-down signals from the FPN to the DMN are crucial to memory encoding (Sestieri et al., 2017). Additionally, prefrontal and parietal circuits that are parts of the FPN are crucial for conscious awareness (Luo et al., 2010; Dehaene and Changeux, 2011; Lapate et al., 2016). Neuroimaging studies in humans identified extinction-related activation in the sensory cortex (Apergis-Schoute et al., 2014; Picó-Pérez et al., 2019). Our results also implicate temporal and sensory cortices, which is consistent with recent animal studies showing that the auditory cortex and sensorimotor regions encode fear memory consolidation (Banerjee et al., 2017; Lai et al., 2018). Collectively, our findings support the engagement of multiple functionally distinct brain networks in fear extinction learning and memory. The results support the need for systems involved in attention control, conscious awareness, and sensory motor to all be engaged in extinction learning and fear regulation, as previously suggested (LeDoux and Pine, 2016).

One novel aspect of our findings is the temporal modification of the large network connectivity that was specific to the conditioned cue. This specific increase in connectivity from early to late extinction learning suggests learning-induced neural plasticity. We are aware of only a few studies that explored temporal patterns of activations within predefined brain regions during fear learning and extinction (Visser et al., 2011; Cole et al., 2013; Braem et al., 2017). And there are emerging studies using network-level analyses to explore emotion processing (McMenamin et al., 2014; Pessoa, 2018; Berg et al., 2020). The activation and connectivity are likely reflecting different mechanisms – the mean brain activation might reflect local processing, whereas brain connectivity may reflect neural plasticity (Mackey et al., 2013; Kelly and Castellanos, 2014) associated with information transfer between distant regions (Bertolero et al., 2015). Consistent with this view, we have observed a dissociation between the connectivity and activation patterns, with larger activation difference (CS+ vs. CS-) in early extinction, but larger connectivity difference in late extinction. We speculate that this large-scale functional connectivity then becomes critical for the consolidation and expression of memory after a delay. In support of this idea, our data show that the magnitude of connectivity changes observed during extinction learning were predictive of the magnitude of extinction memory recall. There are a large number of animal studies in the field of fear conditioning and extinction that support this view (Quirk and Mueller, 2008; Orsini and Maren, 2012; Luchkina and Bolshakov, 2019). For example, fear extinction modulates long-range connectivity between the amygdala and prefrontal regions (Senn et al., 2014), which is critical in the formation of extinction memories (Bukalo et al., 2015).

Following previous studies, we characterized the role of each region by using the regional connectivity measure (Cole et al., 2013; Tagliazucchi et al., 2016). This metric summarizes connectivity between one region and the rest of the brain, which reflects the important role of a region in integrating brain activity in order to coordinate cognition and behavior (Cole et al., 2010). From the view of graph theory, these regions with high regional connectivity measures act as hubs interconnecting distinct, functionally specialized systems (van den Heuvel and Sporns, 2013). Unlike the typical method that testing the region-to-region pairing, the regional connectivity approach requires one statistical test per region, which substantially reduce multiple comparisons and increase the sensitivity in detecting differences between conditions (Cole et al., 2010; Preller et al., 2018). The regional connectivity measure is widely used in resting-state and task-based functional connectivity studies to examine region characteristics (Buckner et al., 2009; Cole et al., 2013; Preller et al., 2018). For example, using similar method, previous studies have shown that regions of the frontoparietal control network rapidly update their pattern of global functional connectivity according to task demands (Cole et al., 2013), and that regional connectivity of the default mode and frontoparietal control network is correlated with consciousness level (Wu et al., 2015). Overall, these studies support that the regional connectivity is a reasonable measure to reflect region characteristics. We found that regions from the DMN, including the precuneus/posterior cingulate cortex and the medial prefrontal cortex, were among the regions that increased their regional connectivity measures the most (Fig. 1A). The increased connectivity with DMN may reflect the demands to integrate the segregated systems to support the complex process during extinction learning. This is consistent with resting-state fMRI studies that suggesting the important role of the DMN for integration of information to facilitate cognitive operations (Buckner et al., 2009; van den Heuvel and Sporns, 2013). We acknowledge that the regional connectivity is a relatively global measure. However, our main results were consistent with the analyses using the more specific network-based statistic analyses (Figure S2), demonstrating the important role of brain regions within the default, frontoparietal, and ventral attention networks in integrating brain activity during extinction learning. In summary, the results from the more specific analyses are consistent with our global analytic approach and still show a robust connectivity changes across many networks that are specific to the conditioned cue, which likely reflects the complex learning that occurs during extinction learning.

Although the SCR-based measure correlates with a subjective report of fear (Hermans et al., 2006; Taschereau-Dumouchel et al., 2019), SCR and ERI may not be ideal measures for conscious feelings of fear (LeDoux, 2014; Taschereau-Dumouchel et al., 2019). Nonetheless, it is important to note that correlation analyses at subnetwork-level revealed that connectivity changes during extinction learning that were most predictive of ERI were within and between the ventral attention, somatomotor, and visual networks. This suggests that changes within these networks related to attention, threat responding, and perception are all important for the consolidation and expression of extinction memory indexed by SCR. Yet, there is still a need to go beyond the ERI measure and develop or test current metrics for conscious awareness of fear and its extinction with functional connectivity changes at the global level. Our results provide partial support to the “two systems” framework by highlighting the contribution of a much larger network of brain regions within the parietal and prefrontal cortices. We acknowledge that without a conscious behavioral measure of fear, it is not possible to test the extent to which neurobiological/physiological responses track with conscious reporting. The novel contribution of this study is that broadly distributed brain regions, especially those from the default mode, frontal-parietal, and ventral attention networks, dynamically changed their functional connectivity during extinction learning, and these connectivity changes are related with the magnitude of extinction memory tested 24 h later. Further studies are needed to examine how such changes in functional connectivity on a large-scale may be linked to, or needed for, conscious awareness and attention control within the context of fear extinction and emotion regulation.

In this study, we used beta-series correlations (BSC) method for task-based functional connectivity estimation. There are other methods that could be used for this estimation, such as the psychophysiological interaction (PPI) or generalized PPI (gPPI) methods (Friston et al., 1997; McLaren et al., 2012; Tompson et al., 2020). We conducted blockwise gPPI analyses on the extinction learning phase as a control for our analytic approach (see Supplemental Material for details). Consistent with our main results, only during the last time-block of extinction learning, we identified 72 brain regions that exhibited larger regional connectivity difference during CS+ processing than CS- processing (Figure S6, *p* < 0.05, FDR-correction). There are differences in detecting context-modulated functional connectivity between gPPI and BSC method (Cisler et al., 2014; Di et al., 2020). Empirical results suggested that BSC is more suitable for event-related design and gPPI is more sensitive in block design (Cisler et al., 2014). Future studies may be conducted to compare the sensitivity of PPI-related and BSC methods in detecting the dynamic changes of task-based functional connectivity.

In summary, our findings provide novel evidence to support recent studies implicating a broader range of brain areas that are engaged in the learning and consolidation of extinction learning. Additional studies are needed to examine how these diffused functional connectivity changes might correlate with, or predict, conscious awareness of the learning. Moreover, studies will be needed to examine whether these functional connectivity changes are preserved or perturbed in clinical populations such as posttraumatic stress disorder and anxiety disorders. Such studies could offer novel targets for neuromodulation within clinical populations that could directly impact threat reactivity, conscious awareness, and the perceptual processes of aversive cues.

## Supplementary Material

1
