# Supplementary material for: Fear extinction learning modulates large-scale brain connectivity
Source: Neuroimage. Author manuscript; Available in PMC 2021 Sep 13. (PMC8436785; doi:10.1016/j.neuroimage.2021.118261)
Supplement: 1 [file NIHMS1730623-supplement-1.docx]

**Supplementary Material**

**Fear Extinction Learning Modulates Large-scale Brain Connectivity**

Zhenfu Wen, Zhe Sage Chen, Mohammed R. Milad

**Network-based statistic analyses**

In addition to the regional connectivity-based analyses in the main text, we conducted the network-based statistic (NBS) analyses to determine network connections that changed during extinction learning (Zalesky, Fornito and Bullmore, 2010). The NBS is a well-validated method for controlling family-wise error rate (FWER). The NBS analysis included the following steps: 1) after obtaining the $432\times432$ connectivity matrixes for CS+ and CS- in extinction learning, we compared each element (edge) of the upper-matrix across subjects (paired t-test, CS+ vs. CS-) in each time-block, and thresholded edges with a p < 0.001 into a set of suprathreshold edges; 2) we identified connected components within the suprathreshold edges, and calculated the sizes of these components; 3) we randomized the CS+ and CS- labels for each participant, and then conducted steps 1 and 2 to estimate the maximal size of connected components, we repeated the procedure 10000 times to obtain null distribution of the size of connected components; 4) we calculated the p value of a component by counting the number of values within the null distribution that were larger than the real component size, and divided it by 10000. A *p* value smaller than 0.05 indicated family-wise error corrected significance of the identified network component.

Across the four time-blocks in extinction learning, we have identified a network component that showed decreased connectivity to CS+ than CS- in early extinction learning (p = 0.031, **Figure S2A**), and identified a network component that showed increased connectivity to CS+ than CS- in late extinction learning (p = 0.007, **Figure S2C**). We grouped the significant connections into within and between subnetwork connections, calculated the number of significant connections in each cell of the subnetwork-level matrix (a symmetric 8 × 8 matrix since we defined 8 subnetworks). In this way, we could better capture the distribution of connections that changed across extinction learning. In early extinction learning (**Figures S2B**), the decreased connectivity to CS+ mainly localized between the visual network and the somatomotor network. While in late extinction learning (**Figures S2D**), the increased connectivity to CS+ mainly localized between the default mode network and other networks, including the somatomotor network, ventral attention network and subcortical network.

**Brain connectivity during CS+U processing**

We also explored the brain connectivity to the CS+ which was not extinguished on day 1 (CS+U, **Figure S3**). We found that the magnitude of mean brain connectivity to the CS+U was higher during the first time-block (early extinction memory recall) relative to the second time-block (late extinction memory recall) (t_135_ = 3.23, p = 0.002). There was no significant difference between CS+E and CS+U (first time-block: t_135_ = -0.05, p = 0.96; second time-block: t_135_ = 0.49, p = 0.62). To test the relevance of change in functional connectivity of the CS+E, CS+U to the magnitude of extinction memory, we averaged the regional connections of the 133 regions across subjects during the last CS+ trial during extinction learning and the first CS+E/CS+U trials during memory recall. We then calculated the similarity between these patterns by using Pearson’s correlation. We found that the pattern of CS+E functional connectivity was more comparable to the connectivity pattern during late extinction learning than CS+U did (Steiger’s Z test, ∆r = 0.12, p < 0.001; CS+E: r = 0.39, p < 0.001; CS+U: r = 0.27, p < 0.001). There are two possible interpretations for these results. The first is that there is some generalization of the extinction-induced changes in functional connectivity to the CS+U, even though there is no significant correlation between CS+U connectivity and connectivity patterns observed in late extinction learning. The second possible explanation is that increased functional connectivity of the CS+U might reflect changes induced by the consolidation and/or retrieval of the conditioned fear memory (given that the CS+U was not extinguished). Our results cannot distinguish between these two possibilities, but future studies perhaps could try to tease these two apart.

**Correlation between ERI and mean connectivity of each subnetwork**

We divided the 432 brain regions into 8 subnetworks as described in the manuscript. We then calculated the mean connectivity difference (CS+ minus CS-) within each subnetwork, or between every two subnetworks, so that we obtained an 8 × 8 symmetric matrix in late extinction learning that represents the subnetwork-level difference. We then calculated the correlation between ERI and each element of the lower triangular part of the 8 × 8 matrix (diagonal elements included). For the CS+E-based ERI, the significant correlations were observed mainly within and between subnetworks of ventral attention, somatomortor, and visual network (**Figure S5**). For the CS+U-based ERI, no significant correlation was found (p < 0.05, FDR-corrected).

We also specifically examined the correlation between ERI and regional connectivity change of amygdala and the hippocampus. For the CS+E-based ERI, we observed positive correlations between extinction-induced amygdala-based connectivity and ERI (r = 0.26, p = 0.005), but no significant correlations were observed within the hippocampus-based connectivity change (r = 0.17, p = 0.07). For the CS+U-based ERI, we did not find significant correlation between it and amygdala-based connectivity change (r = 0.17, p = 0.09) or hippocampus-based connectivity change (r = 0.14, p = 0.15).

**Generalized Psychophysiological Interaction analyses**

We used an alternation method – generalized psychophysiological interaction (gPPI) – to explore the connectivity between brain regions across extinction learning (Friston *et al.*, 1997; McLaren *et al.*, 2012; Tompson *et al.*, 2020). We divided all trials in extinction learning into 4 time-blocks of tasks (4 trials of CS+, 4 trials of CS- in each time-block). For each pair of regions i and j, we constructed a multiple regression model, with the time series of region i as the dependent variable, and the time series of region j, task regressors, and the interaction terms of each task as independent variables. For the interaction term of each task, we first deconvolved the time series of region j with canonical HRF function from SPM12. And then, the deconvolved time series were multiplied by the vector of condition on time of each task. The resulted vectors were re-convolved with the canonical HRF function to obtain the interaction terms for all tasks. Motion parameters were added into the model. After estimation, the beta weight for the interaction term of a specific task was transformed to z-value to represent the connectivity between i and j during the specific task. For CS+/CS- in each time-block, we constructed a 432 × 432 connectivity matrix for all combination of i and j. Because the matrix was not exactly symmetric, we symmetrized the matrix by averaging the upper and lower triangles. We then calculated the regional connectivity as in our main analyses, and compared CS+ vs. CS- in each time-block.

Similar as in our main analyses, in late extinction learning (the 4^th^ time-block), the gPPI method identified 72 brain regions that exhibited larger regional connectivity during CS+ processing than CS- processing (p < 0.05, FDR-based correction). The brain map of these significant regions was shown in **Figure S4**. Comparing with **Figure 1A**, we find that these maps match well, both highlighting the prefrontal cortex and precuneus/posterior cingulate cortex. No significant regions were identified in other time-blocks. These results are consistent with those obtained using the beta series correlations method, suggesting that the functional connectivity of CS+ were increased during late extinction learning.

**References**

Friston, K. J. *et al.* (1997) ‘Psychophysiological and Modulatory Interactions in Neuroimaging’, *NeuroImage*, 6(3), pp. 218–229. doi: 10.1006/nimg.1997.0291.

McLaren, D. G. *et al.* (2012) ‘A generalized form of context-dependent psychophysiological interactions (gPPI): A comparison to standard approaches’, *NeuroImage*, 61(4), pp. 1277–1286. doi: 10.1016/j.neuroimage.2012.03.068.

Tompson, S. H. *et al.* (2020) ‘Functional brain network architecture supporting the learning of social networks in humans’, *NeuroImage*, 210, p. 116498. doi: 10.1016/j.neuroimage.2019.116498.

Zalesky, A., Fornito, A. and Bullmore, E. T. (2010) ‘Network-based statistic: Identifying differences in brain networks’, *NeuroImage*, 53(4), pp. 1197–1207. doi: 10.1016/j.neuroimage.2010.06.041.

**Figure S1.** **The two-day fear conditioning and extinction paradigm.** In day 1, the participants underwent the fear conditioning phase and the extinction learning phase. In day 2, the participants came back to undergo the extinction memory recall phase.


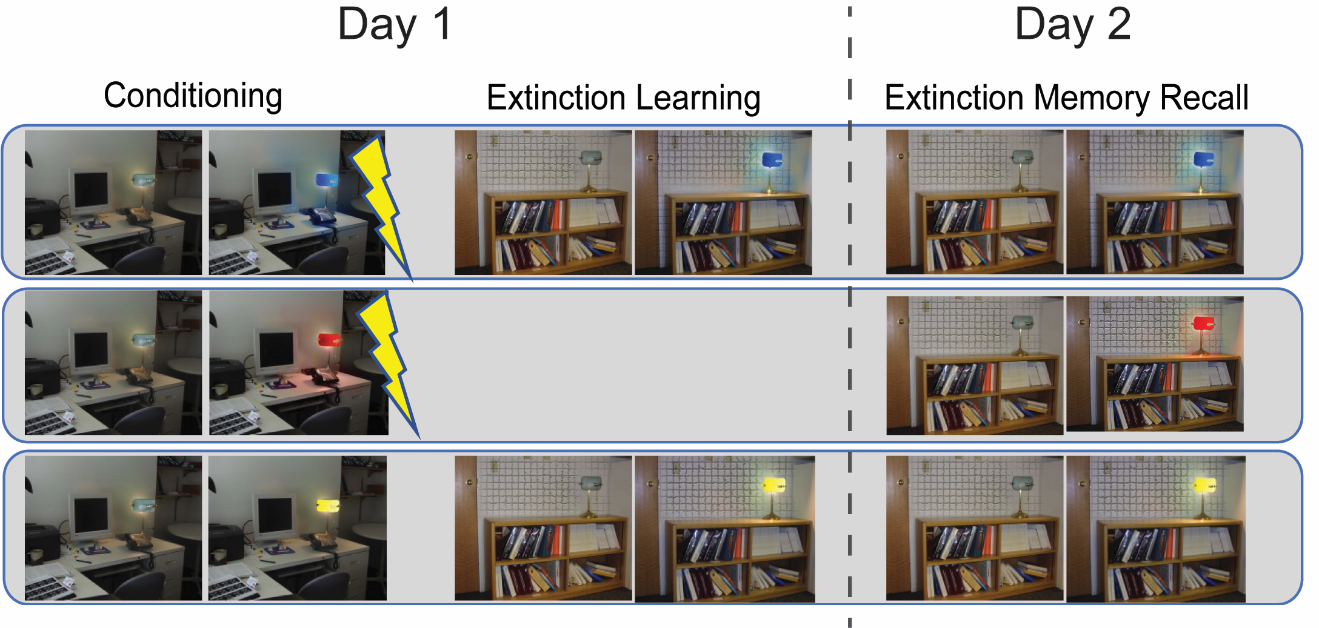


**Figure S2. Connections are significantly different during CS+ and CS- processing in early and late extinction learning. A.** The connections that are significantly lower during CS+ than CS- processing in early extinction learning. The size of each node is proportional to the number of significant connections between this node and other nodes. **B.** The subnetwork-level matrix that represents the distribution of significant connections in early extinction learning. Each element of the matrix means the number of significant connections. **C.** The connections that are significantly higher during CS+ than CS- processing in early extinction learning. **D.** The subnetwork-level matrix that represents the distribution of significant connections in late extinction learning. FPN, frontoparietal control network; DMN, default mode network; DAN, dorsal attention network; LMN, limbic network; VAN, ventral attention network; SMN, somatomotor network; SCN, subcortical network; VN, visual network.


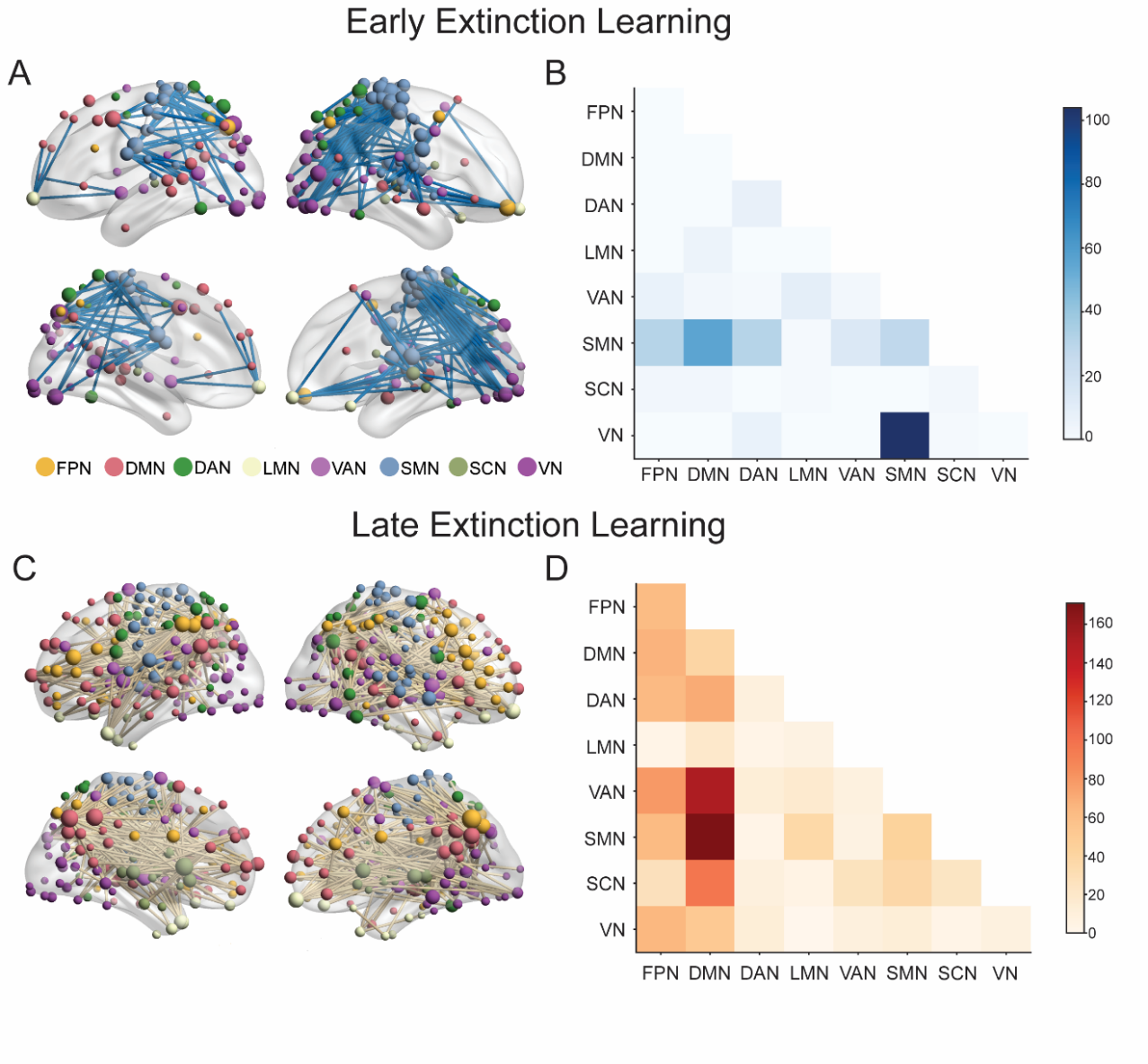


**Figure S3. Mean connectivity of CS+E, CS+U, and CS- during memory recall.** *: p < 0.05, **: p < 0.01.


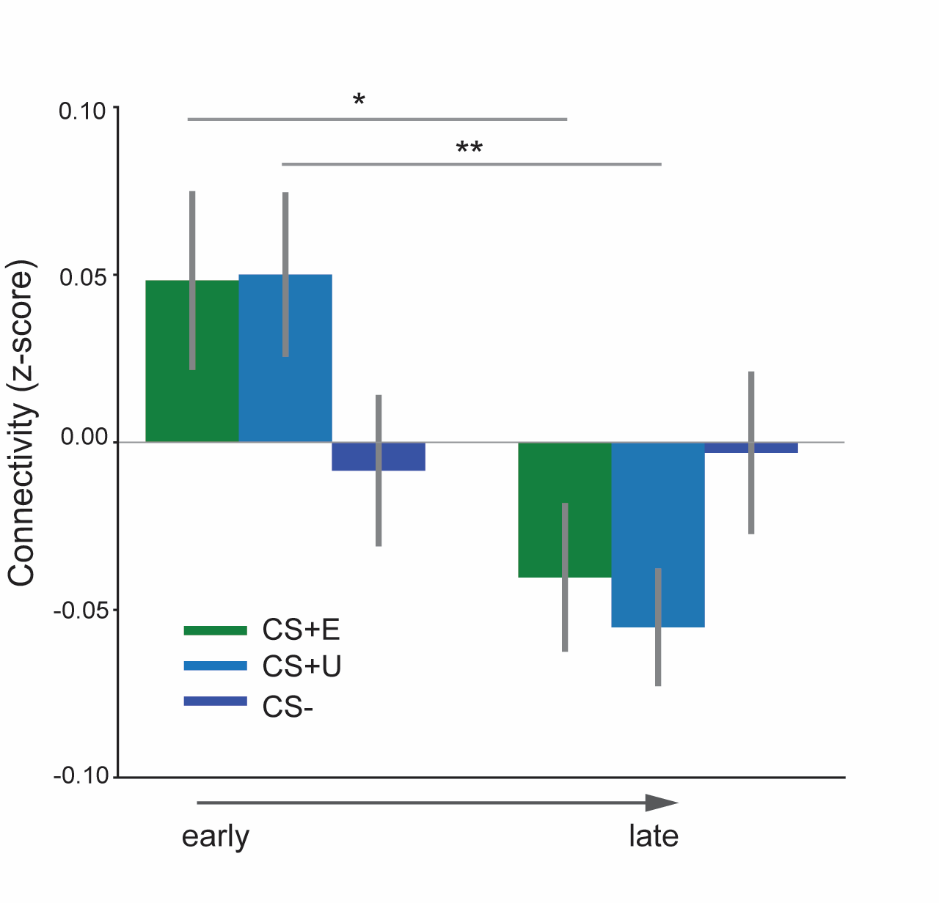


**Figure S4. Percentage of significant regions showing increased connectivity during late extinction learning within each subnetwork.** We have partitioned the whole-brain into 8 functionally distinct subnetworks, including the frontoparietal network (FPN), the default mode network (DMN), the dorsal attention network (DAN), the limbic network (LMN), the ventral attention network (VAN), the somatomotor network (SMN), the subcortical network (SCN), and the visual network (VN).


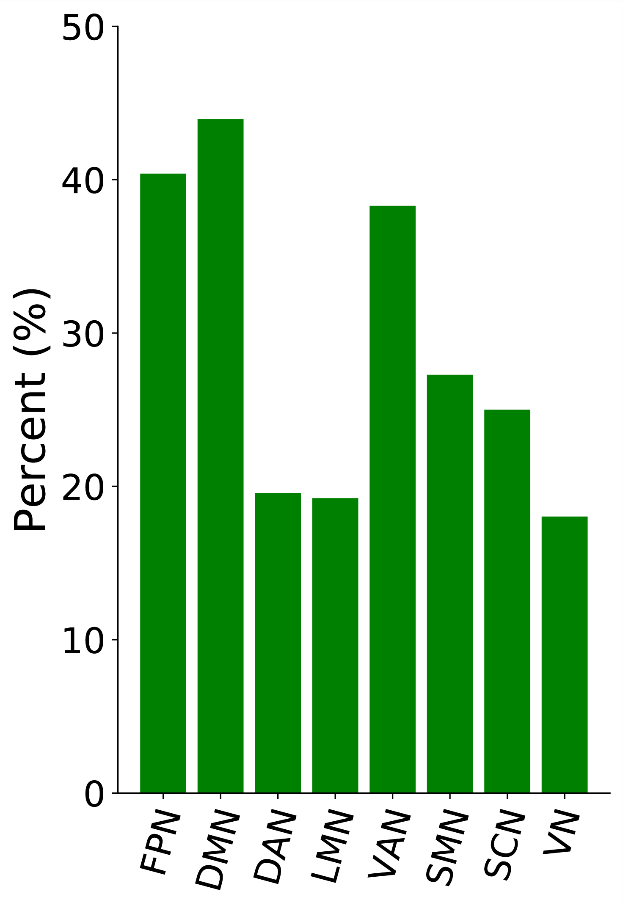


**Figure S5. Correlation between mean connectivity of each subnetwork and the extinction retention index. A.** Correlation between the extinction retention index (ERI) and the mean brain connectivity difference for each subnetwork. **B.** Correlation between the CS+U-based ERI and the mean brain connectivity difference for each subnetwork. Colored tiles represent all significant correlations (p < 0.05, uncorrected; *p < 0.05 FDR-corrected). FPN, frontoparietal control network; DMN, default mode network; DAN, dorsal attention network; LMN, limbic network; VAN, ventral attention network; SMN, somatomotor network; SCN, subcortical network; VN, visual network.


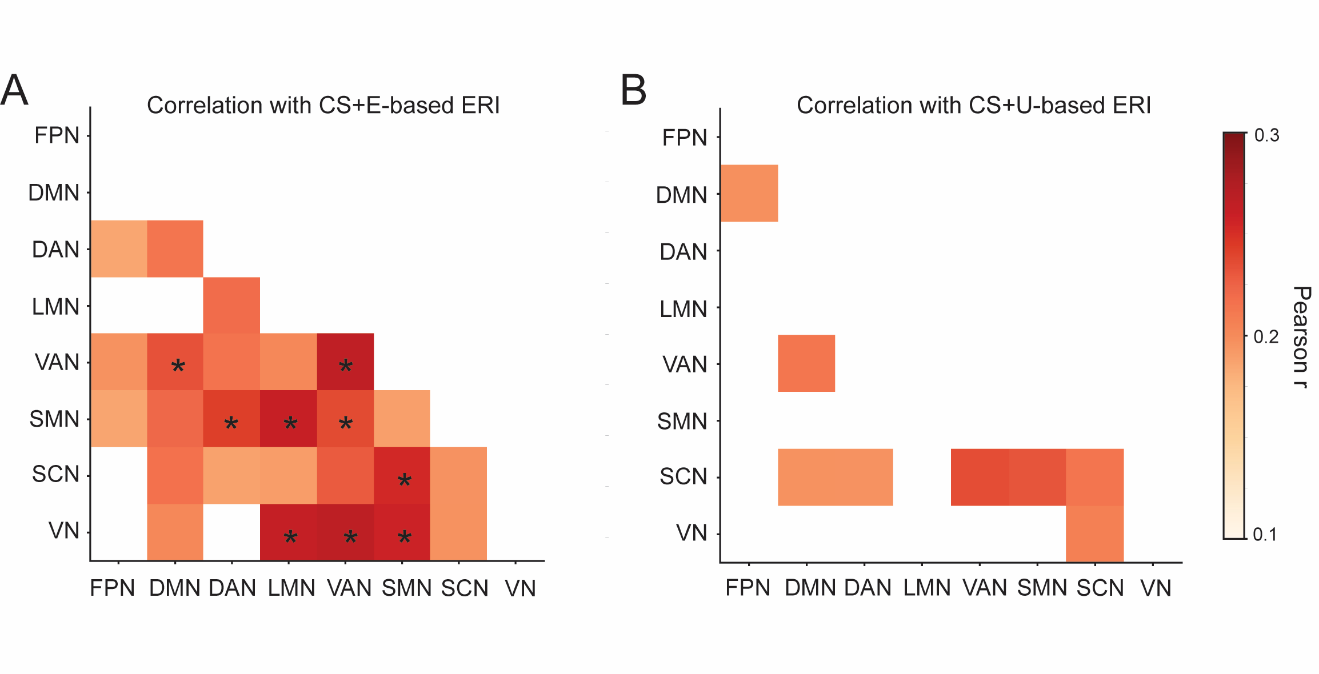


**Figure S6. Brain regions exhibited significant difference in gPPI-estimated connectivity (CS+ minus CS-) during late extinction learning.**


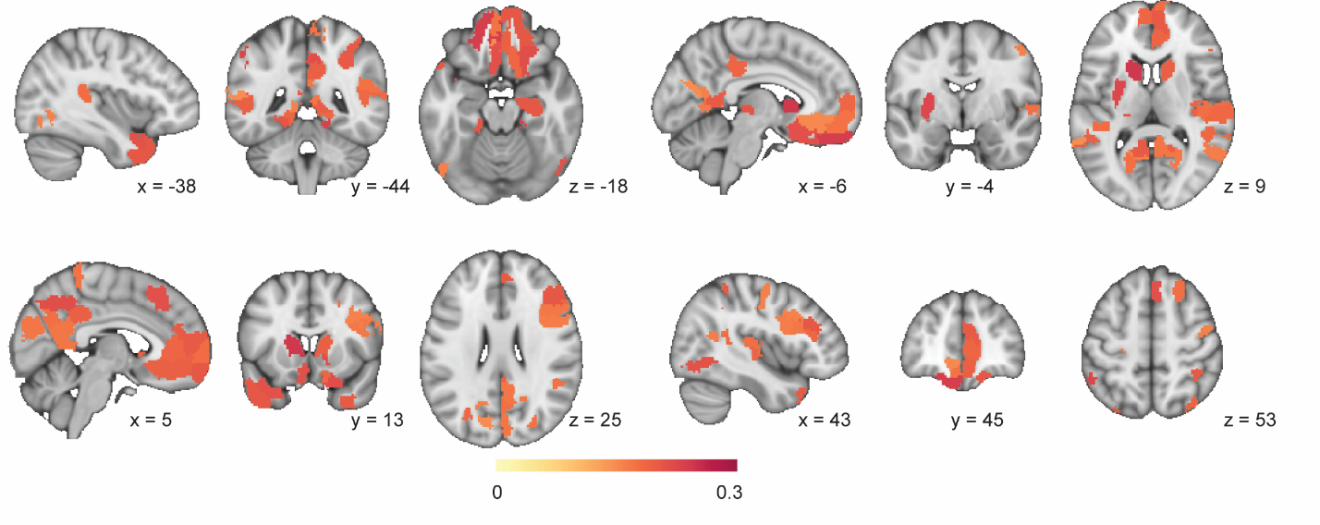


**Table S1. Brain regions that showed significantly higher regional connectivity during CS+ processing than CS- in late extinction learning.**

| **Regions^a^** | **Connectivity^b^  (z-score)** | **Regions** | **Connectivity  (z-score)** |
| --- | --- | --- | --- |
| L Caudate | 0.161 | R Thalamus | 0.12 |
| R Caudate | 0.151 | L Superior Medial Frontal | 0.119 |
| R Precuneus | 0.148 | L Lingual | 0.119 |
| L Inferior Parietal | 0.141 | R Middle Frontal | 0.119 |
| R Posterior Cingulate | 0.14 | R Medial Orbital | 0.118 |
| R Posterior Superior Temporal | 0.138 | R Pallidum | 0.116 |
| L Cuneus | 0.131 | L Rolandic Operculum | 0.116 |
| L SupraMarginal | 0.129 | R Medial Orbital Frontal | 0.116 |
| R Inferior Frontal, Opecular Part | 0.128 | R Middle Cingulate | 0.115 |
| L Middle Temporal Pole | 0.126 | L Calcarine | 0.115 |
| L Middle Frontal | 0.125 | R inferior Parietal | 0.114 |
| L Inferior Frontal, Triangular Part | 0.125 | R Lingual | 0.113 |
| L Putamen | 0.125 | L Superior Frontal | 0.113 |
| R Middle Occipital | 0.124 | R Superior Frontal, Medial | 0.112 |
| R Inferior Frontal, Triangular Part | 0.124 | R SupraMarginal | 0.111 |
| L Heschl | 0.124 | R Superior Frontal | 0.111 |
| R Superior Temporal | 0.123 | R Posterior Cingulate | 0.109 |
| R Calcarine | 0.123 | L Posterior Orbital Frontal | 0.109 |
| L Superior Temporal | 0.123 | L Precentral | 0.108 |
| R Anterior Cingulate | 0.122 | R Cuneus | 0.107 |
| L Middle Temporal | 0.122 | L Thalamus | 0.106 |
| L Precuneus | 0.122 | R Postcentral | 0.105 |
| R Angular | 0.122 | L Supplementary Motor Area | 0.102 |
| R Middle Temporal | 0.122 | R Superior Parietal | 0.101 |
| R Rolandic Operculum | 0.122 | C Inferior Temporal | 0.099 |
| L Insula | 0.122 | L Middle Cingulate | 0.097 |
| L Postcentral | 0.122 | L Superior Parietal | 0.095 |
| R Insula | 0.122 | R Supplementary Motor Area | 0.095 |
| L Anterior Cingulate | 0.121 | R Rectus | 0.084 |

a: The region name was defined based on the automated anatomical labelling (AAL) atlas. All of the 133 significant regions identified in late extinction learning were assigned to 58 AAL regions as listed.

b: The connectivity values were mean regional connectivity (CS+ minus CS-) across all regions that were assigned the AAL label.

L: Left; R: Right
